# Supplementary material for: Comparison of Two Electronic Physical Performance Batteries by Measurement Time and Sarcopenia Classification
Source: Sensors (Basel). 2021 Jul 29;21(15):5147. doi: 10.3390/s21155147 (PMC8347973; doi:10.3390/s21155147)
Supplement: Supplementary file 1 [file sensors-21-05147-s001.zip › sensors-1267136-SI.pdf]

sensors

Supplementary Material

Comparison of Two Electronic Physical Performance Batteries by Measurement Time and Sarcopenia Classification

Chan Mi Park [1,2], Hee-Won Jung [1,*], Il-Young Jang [1,*], Ji Yeon Baek [1], Seongjun Yoon [3], Hyunchul Roh [3] and Eunju Lee [1]

[1] Asan Medical Center, Division of Geriatrics, Department of Internal Medicine, University of Ulsan College of Medicine, Seoul 05505, Korea; chanmipark.paper@gmail.com (C.M.P.); dreamcatch899@gmail.com (J.Y.B.); eunjulee@amc.seoul.kr (E.L.)
[2] Harvard T.H.Chan School of Public Health, Boston, MA 02115, USA
[3] Dyphi Research Institute, Dyphi Inc., Daejeon 34068, Korea; seongjun@dyphi.com (S.Y.); roh@dyphi.com (H.R.)
* Correspondence: dr.ecsta@gmail.com (H.-W.J.); onezero2@gmail.com (I.-Y.J.);
Tel.: +82-2-3010-1852 (H.-W.J.); +82-2-3010-1658 (I.-Y.J.)

Citation: Park, C.M.; Jung, H.-W.; Jang, I.-Y.; Baek, J.Y.; Yoon, S.; Roh, H.; Lee, E. Comparing Two Electronic Physical Performance Batteries by Measurement Time and Sarcopenia Classification. *Sensors* **2021**, *21*, 5147. https://doi.org/10.3390/s21155147

Academic Editor: Adamantios Arampatzis

Received: 3 June 2021
Accepted: 22 July 2021
Published: 29 July 2021

Publisher's Note: MDPI stays neutral with regard to jurisdictional claims in published maps and institutional affiliations.

Copyright: © 2021 by the authors. Submitted for possible open access publication under the terms and conditions of the Creative Commons Attribution (CC BY) license (http://creativecommons.org/licenses/by/4.0/).

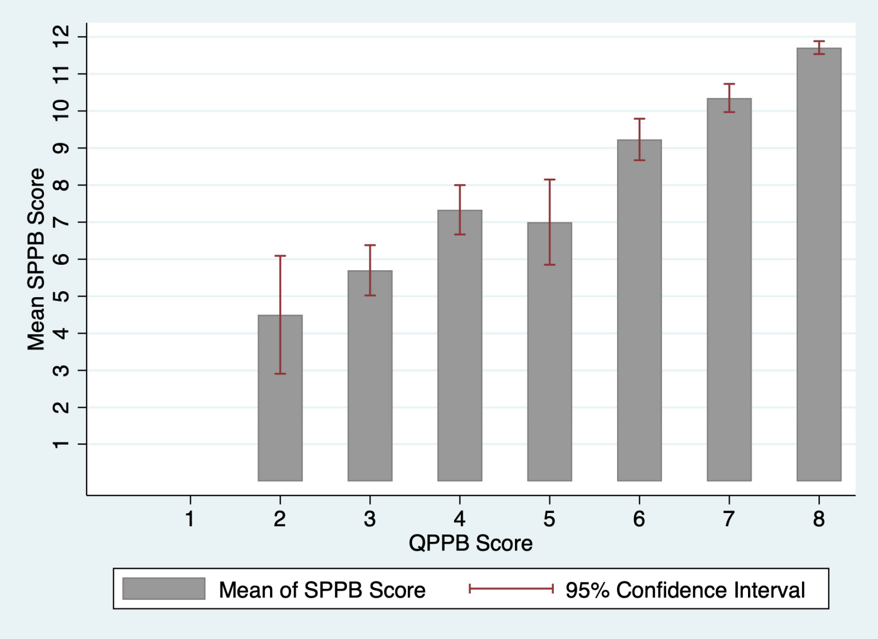

Figure S1. Distribution of electronic Short Physical Performance for corresponding electronic Quick Physical Performance Battery.
